# Supplementary material for: Broadscale dampening of uncertainty adjustment in the aging brain
Source: Nat Commun. 2024 Dec 23;15:10717. doi: 10.1038/s41467-024-55416-2 (PMC11666723; doi:10.1038/s41467-024-55416-2)
Supplement: Supplementary file 1 — Supplementary Information [file 41467_2024_55416_MOESM1_ESM.pdf]

Supplementary Information for

Broadscale dampening of uncertainty adjustment in the aging brain

Julian Q. Kosciessa*, Ulrich Mayr, Ulman Lindenberger, & Douglas D. Garrett*

* Email: kosciessa@mpib-berlin.mpg.de; garrett@mpib-berlin.mpg.de

This PDF file includes:

Supplementary Figures 1 to 18
Supplementary Notes 1 to 13
Supplementary Table S1-5
Supplementary References

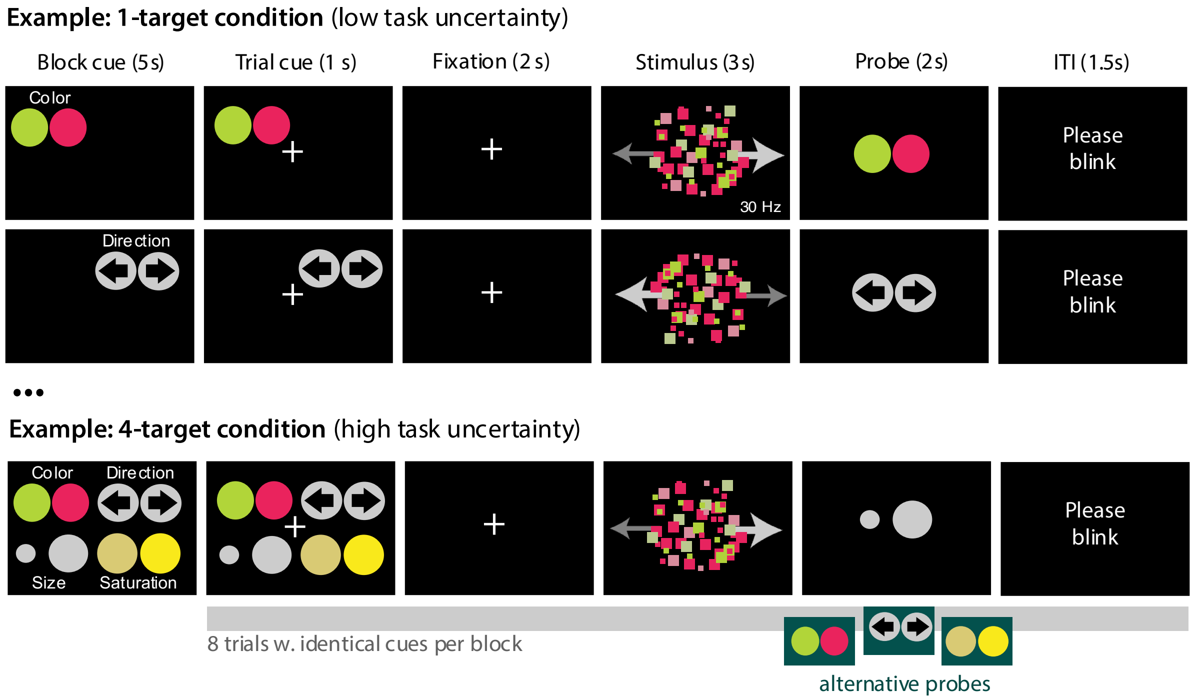

Supplementary Figure 1. Examples of design phases. The top illustrates a trial sequence of the low uncertainty condition. Here, a single feature cue provides specific advance information about the upcoming probe. The bottom illustrates a trial sequence for the maximum uncertainty condition, in which all four features are cued in parallel. Amongst the cued features, a single feature will be selected as probe with equal probability. For all conditions, the cue remains identical for eight subsequent trials (= a cue block). Feature cues and probes are indicated by their respective icons.

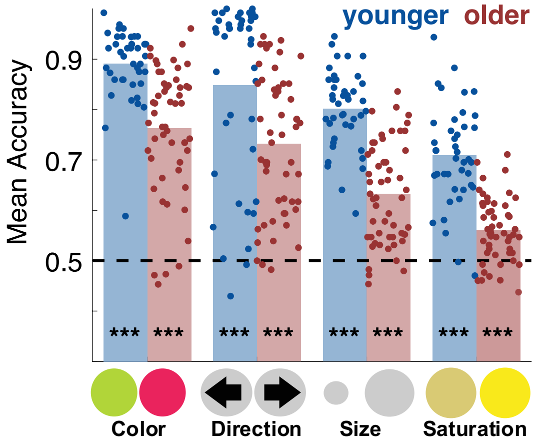

Supplementary Figure 2. Average accuracy across uncertainty conditions. Younger (N = 42) and older (N = 53) adults on average performed the task above chance for all features that were probed. Statistics are based on one sample t-tests against chance level (0.5 in this 2AFC task). Estimates are averaged across EEG and fMRI sessions. *** p < .001.

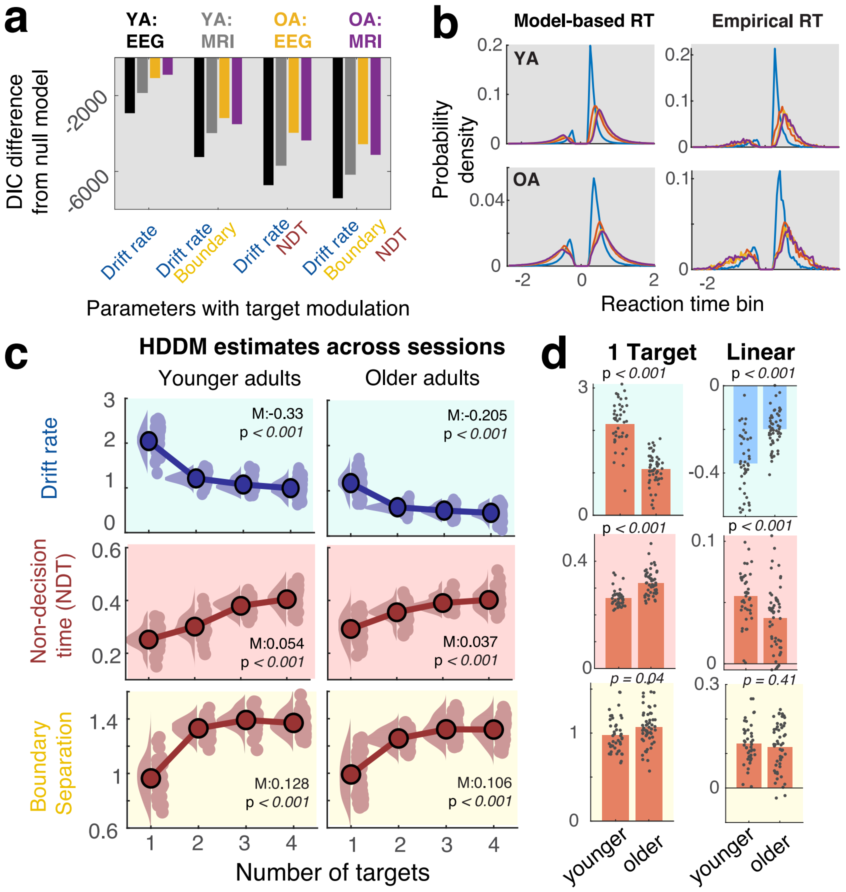

Supplementary Figure 3. Age-related uncertainty adjustments to decision processes. (a) DIC-based model comparison indicates that a model, including uncertainty modulation of drift rates, non-decision times, and boundary separation provides the best group fit to the behavioral data. (b) Posterior predictive checks for the full model (shown for the EEG session). Negative RTs indicate incorrect responses. Model-based ("posterior predictive") values were sampled 50 times within each participant and condition (as implemented in the HDDM package), and probability density (100 RT bins) was estimated first within-subject across all samples, and then averaged across participants. In empirical data, probability densities were estimated across all participants due to the sparse within-subject RT counts. (c) Uncertainty modulation of HDD parameter estimates, averaged across sessions. Statistics refer to paired t-tests of linear slopes against zero. Data are within-subject centered. (d) Age comparison of single-target parameter estimates (left) and linear uncertainty effects (~age x target load interaction). Statistics refer to unpaired t-tests. $N_{younger} = 42$, $N_{older} = 53$.

Supplementary Note 1

Uncertainty and age effects on non-decision time and boundary separation. The main analyses targeted drift rate as the main parameter of interest. Given that the best-fitting model (Supp. Fig. 3ab) included uncertainty variation also for non-decision times as well as boundary separation, we explored the potential variation of the latter two parameters with age and uncertainty (Supp. Fig. 3c). In contrast with younger adults, older adults had significantly longer non-decision times, and larger boundary separation, suggesting that more evidence was collected prior to committing to a choice. There is some evidence from 2AFC tasks that older adults adopt decision boundaries that are wider than the boundaries of younger adults [1,2] [but see [3]], which may signify increased response caution. In both age groups, we observed uncertainty-related increases in non-decision times, albeit more constrained in older adults, as well as similar increases in boundary separation as a function of rising uncertainty (see Supp. Fig. 3d). Notably, the uncertainty effect on boundary separation was not consistently reproduced by either the integration threshold of the domain-general CPP (Supp. Fig. 6b), or the effector-specific contralateral beta power threshold (Supp. Fig. 7d), highlighting uncertainty regarding the true effect on behavioral response caution, or neural proxy signatures thereof. These discrepancies deserve further attention in future work and may suggest that a model with alternative parameter constellations could provide a more coherent description. Convergence of the current model with our previous results in younger adults [4] ultimately argues for robust drift rate inferences that were independent from the specific model choice.

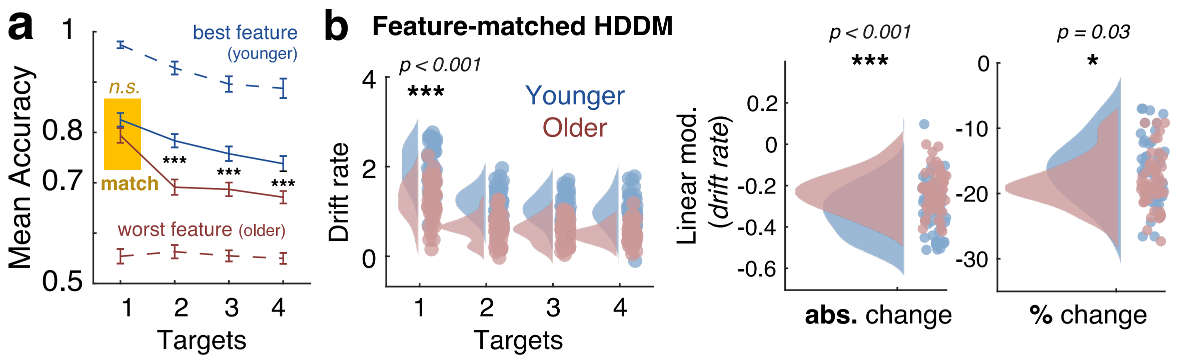

Supplementary Figure 4. Drift rate differences do not arise from accuracy ceiling or floor effects. (**a**) Difficulty with selectively distinguishing individual features is a major component of the task, which may contribute to age differences in single-target drift rates. While on average, younger adults' single-target responses were more accurate than those of older adults, this differed between features (see Supp. Fig. 2). Excluding the most accurate ('best') feature for younger adults and the least accurate ('worst') feature for older adults matched groups regarding their accuracy in the single-target condition. In this scenario, older adults showed more pronounced accuracy decreases under uncertainty, relative to younger adults. Data are means +/- SEs and include data from EEG and fMRI sessions ($N_{younger}$ = 42, N_{older} = 53). n.s.: p = 0.13; ***1: p = 2.6e-05; ***2: p = 6.1e-04; ***3: p = 8.2e-04. (**b**) Drift rate estimates for an HDDM model that only included age-matched features ("match" in ***a***). This model indicated retained age differences in single-target drift rate and absolute drift changes under uncertainty (right). Older (vs. younger) adults showed stronger *relative* drift rate reductions from the single-target baseline.

Supplementary Note 2

Drift rate effects for accuracy-matched features. Our analysis indicated that older adults on average showed reduced behavioral uncertainty costs. However, these uncertainty costs are thought to arise from attending to a varied feature set, whose discrimination also varies between age groups when only a single feature is relevant. To examine whether potential ceiling or floor effects in feature-specific accuracy (e.g., due to varying perceptual uncertainty) acts as a between-group confound, we sorted features according to their single-target accuracy in each participant, and averaged accuracy according to such "preference" within each age group. This revealed that three out of the four features elicited comparable single-target accuracy between age groups, whereas only the best feature of younger adults, and the worst feature of older adults could not be matched (Supp. Fig. 4a). To test the robustness of unmatched drift rate estimates (Fig. 1b), we created HDDM models that excluded the most preferred feature of younger adults, and the individually least preferred feature in older adults (i.e., only including "matched" features). Results from this control analysis are shown in Supp. Fig. 4b. We observed retained age differences in single-target drift rates, as well as uncertainty-related drift rate changes that mirrored our main results. These results indicate that baseline feature differences are likely not the principal origin of age and uncertainty drift rate differences.

Supplementary Note 3

More pronounced relative performance decreases in older adults. Compared with younger adults, older adults' drift rates were lower across levels of target load (Fig. 1b). To test whether drift rates across all set sizes show similar proportional age changes, we calculated relative drift rate changes. Arguing against uncertainty-independent age differences in drift rate, we observed larger *relative* drift rate decreases under uncertainty in older as compared with younger adults (see Supp. Fig. 4b right for feature-matched HDDM; similar results were obtained in the main model). This indicates that despite being smaller in absolute terms, older as compared to younger adults suffered stronger *relative* drift rate losses once uncertainty was introduced. This mirrored larger accuracy decreases in matched features once uncertainty was introduced (Supp. Fig. 4a). Taken together, this indicates that uncertain multi-tasks contexts present an outsized challenge to older adults' performance, over and above challenges in single-target specificity [5]. For our main analyses that target inter-individual relations, we focus on absolute uncertainty-related drift rate changes due to their relation to neural uncertainty adjustment in prior work (Kosciessa et al., 2021), and the computational interpretability of absolute drift rates at each target load.

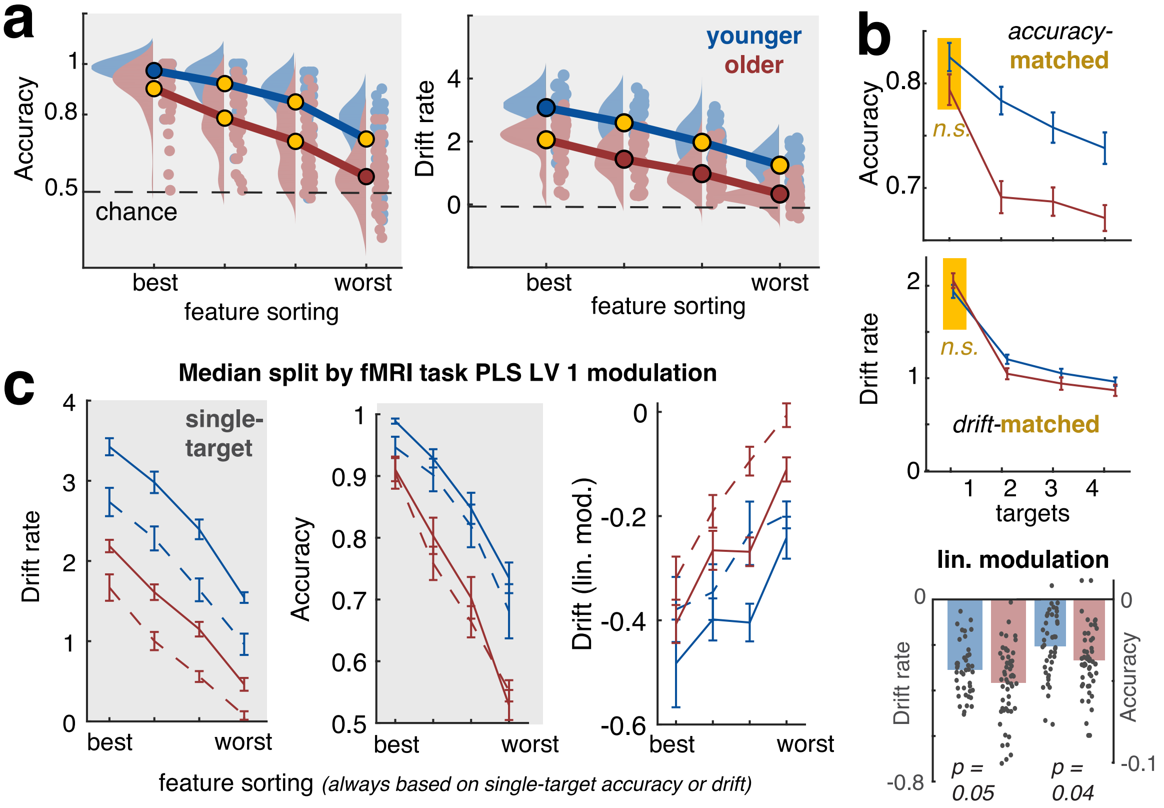

Supplementary Figure 5. Exploration of feature-specific accuracy and drift rate variation. (a) Single-target accuracy and drift rate for features that are sorted by decreasing single-target accuracy and drift rate. Yellow dots indicate the conditions selected for matching in b. (b) Matching based on single-target accuracies and drift rates indicates stronger *relative* performance decreases with uncertainty in older compared with adults. (c) A median split of fMRI Brainscores (trichotomized split | full lines: largest BOLD mod.; dashed lines: smallest BOLD mod.; cf. Supp. Fig. 17) indicates that larger neural modulation is inter-individually and group-wise linked to higher single-trial drift rates (but not differential accuracy; see also Supp. Fig. 4), independent of feature preference. This suggests that neural modulation is not primarily constrained due to varying choice difficulty for individual features, but rather by a global operation (e.g., cue-guided selectivity, distractor suppression, etc.) that is shared across features. Means +- SEMs ($N_{younger}$ = 42, N_{older} = 53).

Supplementary Note 4

Exploration of feature-specific accuracy and drift rate variation. We performed exploratory analyses to further characterize behavior (accuracy, rt) and drift rate as a function of probed feature. To this end, we estimated all canonical HDDM parameters also for variations in the feature that has been probed. We caution that the large parameter space may lead to some convergence issues, which is why the main model does not estimate parameters for separate features. We sorted single-target accuracy and drift rate in descending order (Supp. Fig. 5a). Like accuracy, selecting features such that single-target drift rates are matching indicates *larger* uncertainty-induced drift rate decreases (Supp. Fig. 4b, see also **Supplementary Note 3**). However, it is debatable whether this is a *reasonable* comparison particularly for drift rates: an analysis that matches "selective" performance (i.e., drift rates under low uncertainty) between age groups also removes key age differences in neural engagement. Namely, we observed a closer link between within-group fMRI modulation and single-target drift rates, but not accuracy (Supp. Fig. 4c, see also Supp. Fig. 3). This was independent of which feature was probed, and thus unrelated to varying perceptual uncertainty between features. This reinforces the notion that perceptual uncertainty per se (which in psychophysics is sometimes titrated to comparable *accuracy* levels) is not the principal predictor of the constrained neural uncertainty modulation either within or between age groups.

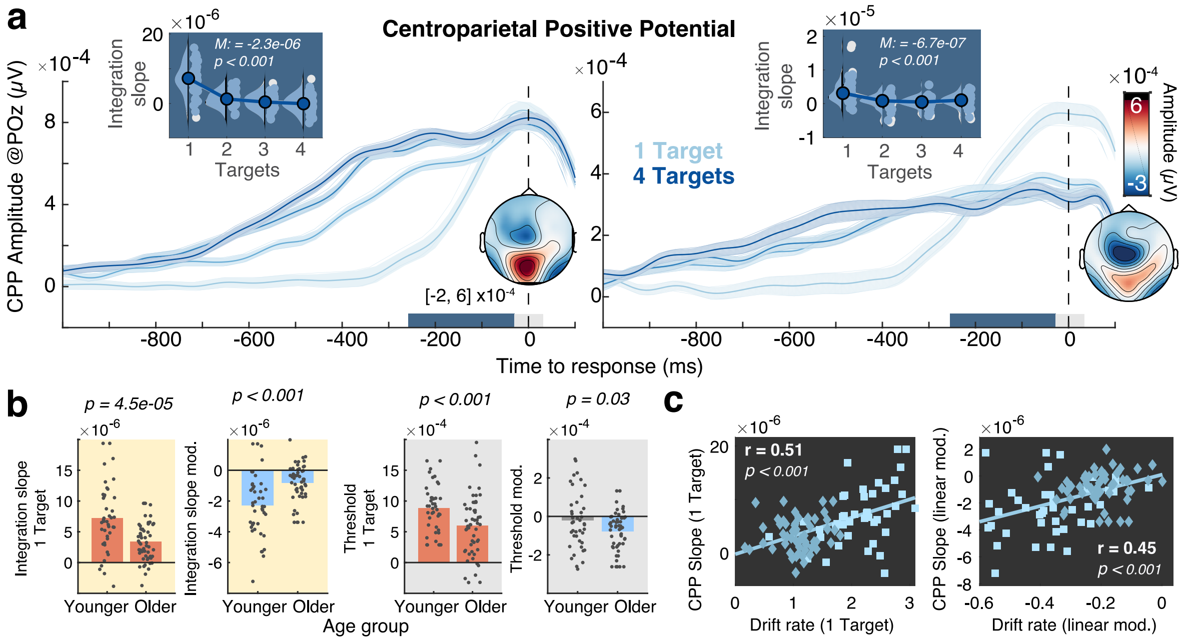

Supplementary Figure 6. Centroparietal Positive Potential (CPP) as a signature of domain-general evidence integration. (a) Modulation of CPP as a neural signature of evidence accumulation (mean ± within-subject SEM). The integration slope of the response-locked CPP decreases with increasing uncertainty. Traces are mean ± within-subject SEM ($N_{younger}$ = 47, N_{older} = 53). Insets show CPP slope estimates from −250 to −50 ms relative to response execution. (b) Age comparison of CPP integration slopes (yellow background) and CPP integration thresholds (grey backgrounds). (c) CPP estimates of evidence integration converge with behavioral drift rate estimates at the interindividual level, both w.r.t the single-target condition ($t(93) = 4.86$, $p < 0.001$, $r = 0.45$, 95%CI = [0.27,0.59]; *age-partialed*: $t(92) = 2.70$, $p = 0.01$ $r = 0.27$, 95%CI = [0.08,0.45) and linear effects of target number ($t(93) = 5.72$, $p < 0.001$, $r = 0.51$, 95%CI = [0.34,0.64]; *age-partialed*: $t(92) = 3.49$, $p < 0.001$, $r = 0.34$, 95%CI = [0.14,0.5]). Squares: younger adults; diamonds: older adults.

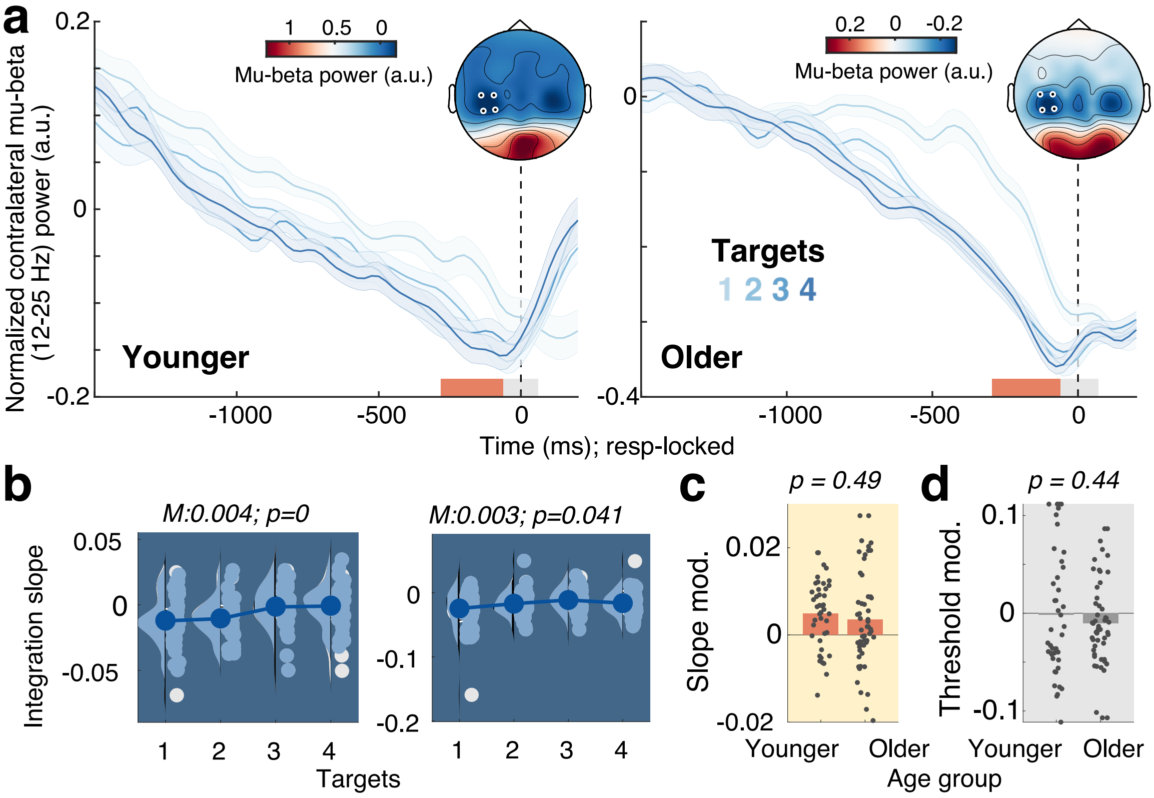

Supplementary Figure 7. Contralateral beta power as a signature of motor-specific response preparation. (a) Pre-response desynchronization of contralateral mu-beta power shallow with increasing number of targets ($N_{younger}$ = 47, N_{older} = 53). Traces show means +/- within-subject SEM. (b) Linear slope estimates, estimated via linear regression from -250 ms to -50 ms, relative to response. Data are within-subject centered for visualization. Statistics refer to paired t-tests of linear slopes against zero. (c, d) Age comparison of linear modulation of beta slopes (c) and integration thresholds (d) by target load. Statistics refer to unpaired t-tests.

Supplementary Note 5

Motor-specific response preparation. In addition to the domain-general CPP, we also investigated motor-specific contralateral beta power (Supp. Fig. 7a). Extending results from behavioral modeling, and CPP integration slopes, we observed a shallowing of pre-response beta power build-up, suggesting decreases in response preparation (Supp. Fig. 7b). However, such shallowing was not statistically different between age groups (Supp. Fig. 7b), thus deviating from the age x load interaction that we observed for the remaining integration signatures. Furthermore, linear changes in beta slope as a function of target load were neither associated with linear drift changes ($t(93) = -0.29$, $p = 0.77$, $r = -0.03$, 95%CI = [-0.23,0.17]) nor CPP slopes ($t(93) = -1.07$, $p = 0.29$, $r = -0.11$, 95%CI = [-0.3,0.09]) across age groups. The parameters were also not directly related in the single-target condition (*drift rates*: $t(93) = 1.76$, $p = 0.07$, $r = 0.18$, 95%CI = [-0.02,0.37]; *CPP slopes*: $t(93) = -0.58$, $p = 0.55$, $r = -0.06$, 95%CI = [-0.26,0.14]). Motor-specific response preparation thus appears to partially dissociate from effector-unspecific evidence integration at the individual level.

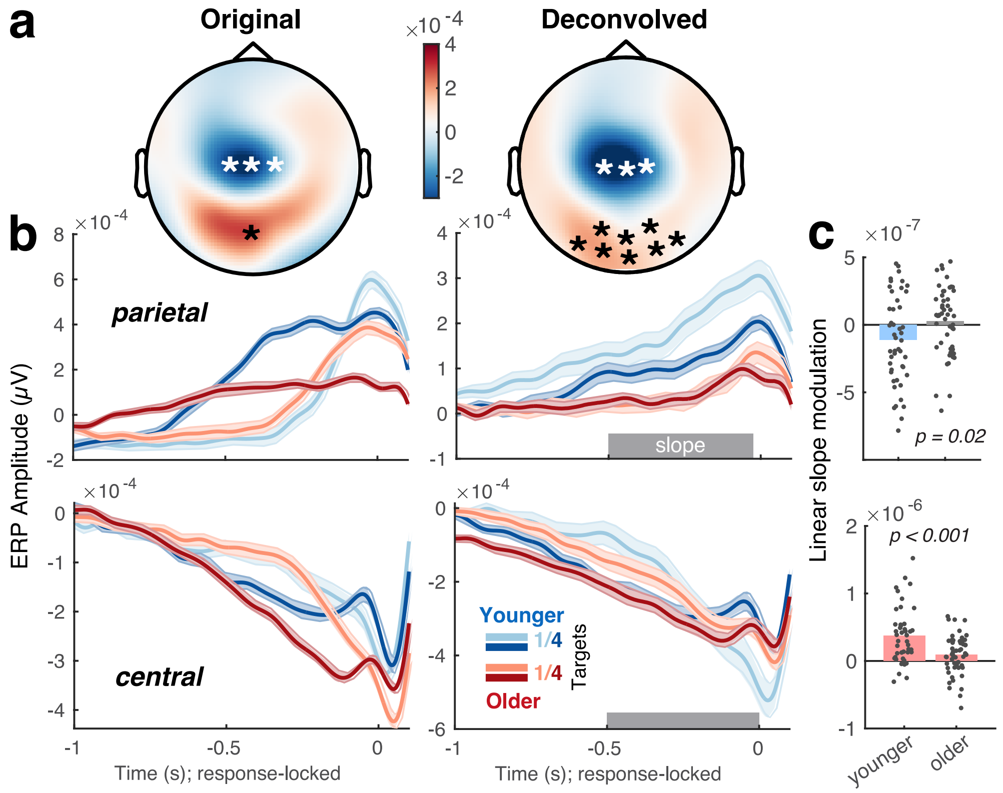

Supplementary Figure 8. Deconvolution analyses for response-locked potentials. (a) Average peri-response topographies from -250 ms to 100 ms before (left, cf. Fig. 1c) and after (right) probe onset deconvolution. Black stars indicate sensors selected for parietal/CPP potentials, whereas white stars indicate central channels selected for motor-related potentials. (b) Response-related potentials before (left) and after (right) probe onset deconvolution. Means +- within-subject SEM ($N_{younger}$ = 47, N_{older} = 53). Parietal (top) and central (bottom) potentials correspond to the channels indicated in a. (c) Uncertainty modulation (linear) of pre-response slopes (deconvolved data; cf. Fig. 1c) by age group. Pre-response slopes were estimated during the time windows indicated by the shaded areas in b.

Supplementary Note 6

Deconvolution of probe-related from response-locked ERP potentials. Recent work proposes that pre-response CPP ramping could be sufficiently explained by a probe-related appraisal process – rather than choice – that is jittered in time due to response speed variations [6]. To explore this here, we conducted deconvolution analyses using the unfold toolbox [7] implemented in MATLAB. By providing event-specific regression formulas, this approach can disentangle temporally overlapping ERP responses. We jointly modelled probe onset and response regressors using FIR basis functions, time expanded +/- 1 seconds around the respective events [6]. Data were 8 Hz lowpass-filtered, no baseline correction was applied, amplitudes > 250 μV were removed. Consistent with the CPP reflecting a stimulus-related P300 potential [8,9], posterior topographies changed following removal of onset-related activity (Supp. Fig. 8). However, parieto-occipital potentials more exhibited residual ramping that peaked slightly prior to response onset, in line with a choice-related process. Age differences in the uncertainty modulation of residual slopes (linear fits: -500 to -50 ms) resembled those in the main analysis (cf. Fig. 1c), albeit with no uncertainty-related slope adjustment observed in older adults. Analogous to beta power dynamics (Supp. Fig. 7), a central motor-related potential indicated that uncertainty shallowed slopes of the deconvolved motor negativity (linear fits: -500 to -0 ms).

While onset deconvolution captures the impact of probe processing on response-locked potentials, it provides inconclusive information regarding an accumulation-to-bound decision process. Differences in evidence integration rate can emerge rapidly after probe onset, persist until response see e.g., [9,10], and produce differential RT distributions [9]. In the face of temporal covariance, considering unique response-locked variance would remove substantial "choice" signal of interest. In the face of this ambiguity regarding the latent processes contributing to observed potentials, multiple observations support a choice-related account here: First, CPP slopes inter-individually mirror model-based (HDDM) estimates of evidence integration (Supp. Fig. 6c). Second, similar ramping dynamics are observed in the motor system (**Supplementary Note 5**, central ERP above), where probe impact is more limited. Notably, contralateral beta power reflects relative biases towards one hemispheric response over the other, thereby fulfilling strict definitions of a choice-specific signature [6]. Effector-specific signatures [11] therefore lend further credence to the notion that our neural signatures capture evolving decision processes.

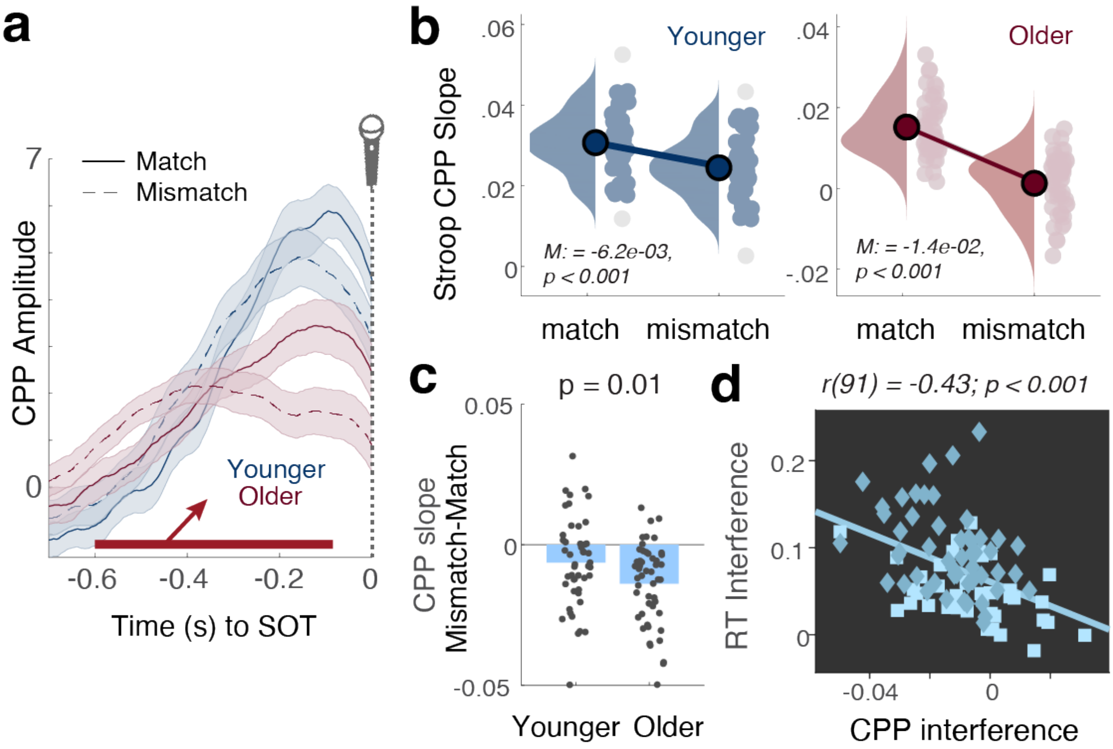

Supplementary Figure 9. CPP slope during the Stroop task. (a) Response-aligned CPP traces split by condition and age group. Time series were smoothed with 60 ms windows for visualization, but not for slope fitting. Linear slopes were estimated during the interval of -600 to -100 ms prior to indicated SOTs, marked by the red line. (a) CPP integration slopes were reduced in magnitude in the mismatch condition in both age groups ($N_{younger} = 47$, $N_{older} = 51$). (b, c) Interference effects on CPP slopes were more pronounced in older compared with younger adults. (d) The magnitude of individual interference effects was similarly reflected in RTs and CPP slopes: individuals with more pronounced RT slowing also exhibited larger CPP slope reductions ($t(91) = -4.54$, $p < 0.001$, $r = -0.43$, 95%CI = [-0.58,-0.25]; partial-correlation accounting for age: $t(90) = -3.22$, $p < 0.001$, $r = -0.32$, 95%CI = [-0.49,-0.12]). Squares: younger adults; diamonds: older adults.

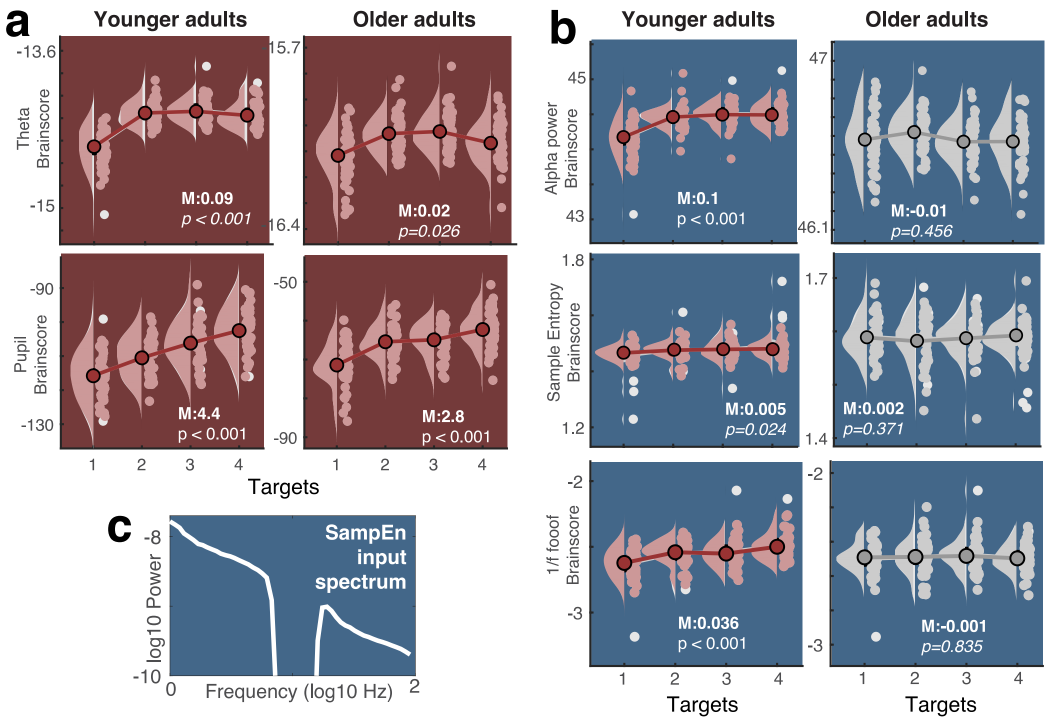

Supplementary Figure 10. Load modulation for cognitive control (a) and excitability signatures (b). Statistics refer to paired t-tests of linear slopes against zero ($N_{younger}$ = 47, N_{older} = 53). In line with the different excitability indices capturing a shared latent characteristic, the magnitude of uncertainty modulation was inter-individually related among the three parameters (alpha-1/f: r = 0.44, p = 9.8e-06; 1/f-SampEn: r = 0.6, p = 1.1e-10; SampEn-alpha: r = 0.24, p = .02). (c) Sample entropy input spectrum highlighting the exclusion of alpha-range signal content.

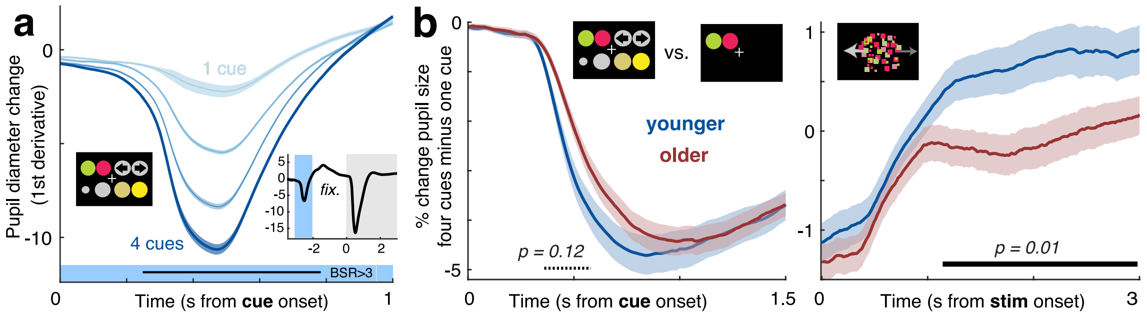

Supplementary Figure 11. Pupil size modulation in response to cue onset. (a) Temporal derivatives of pupil diameter linearly decreased with increasing number of feature cues. Data are means +- within-subject SEM across age groups ($N_{younger}$ = 47, N_{older} = 53). The black line indicates significant loadings of a Task PLS LV1 (*permuted* p ~ 0). The inset shows grand average pupil dynamics with shaded cue and stimulus phases. (b) Relative pupil constriction upon presentation of more cues (= increasing luminance) did not differ between age groups, whereas younger adults showed larger relative pupil size for (luminance-matched) stimuli in the face of more uncertain feature relevance than older adults.

Supplementary Note 7

Stimulus-related pupil modulation does not result from cue-related luminance differences. The task cue is composed of one to four bright exemplars. It thereby linearly increases in luminance alongside our uncertainty manipulation. To test whether luminance changes in the cueing phase affected stimulus-related pupil modulation, we probed pupil dynamics that were time-locked to cue onset (Supp. Fig. 11a). Upon cue onset pupil size linearly decreased, in line with increasing luminance. These changes were however constrained to the cue presentation interval. Uncertainty-related pupil size changes upon stimulus presentation thus cannot be attributed to luminance differences in the preceding cue. Complementing the perspective on the 1st derivative of pupil dynamics, we also probed relative pupil size changes (calculated as % change relative to a condition-specific 500 ms baseline prior to cue onset: *data-baseline)/baseline*). Changes between max. and min. target load (= maximal cue luminance differences) are shown by age group in Supp. Fig. 11. Age groups did not significantly differ in relative pupil constriction upon cue presentation (~ luminance-effect; indicated by a cluster-based permutation test), but younger adults showed relatively increased pupil size in the face of more uncertain feature relevance following the onset of (luminance-matched) stimuli. These results jointly indicate that stimulus-related age differences in uncertainty-related pupil size modulation (shown in Fig. 4b) are not merely a spill-over of differential luminance sensitivity.

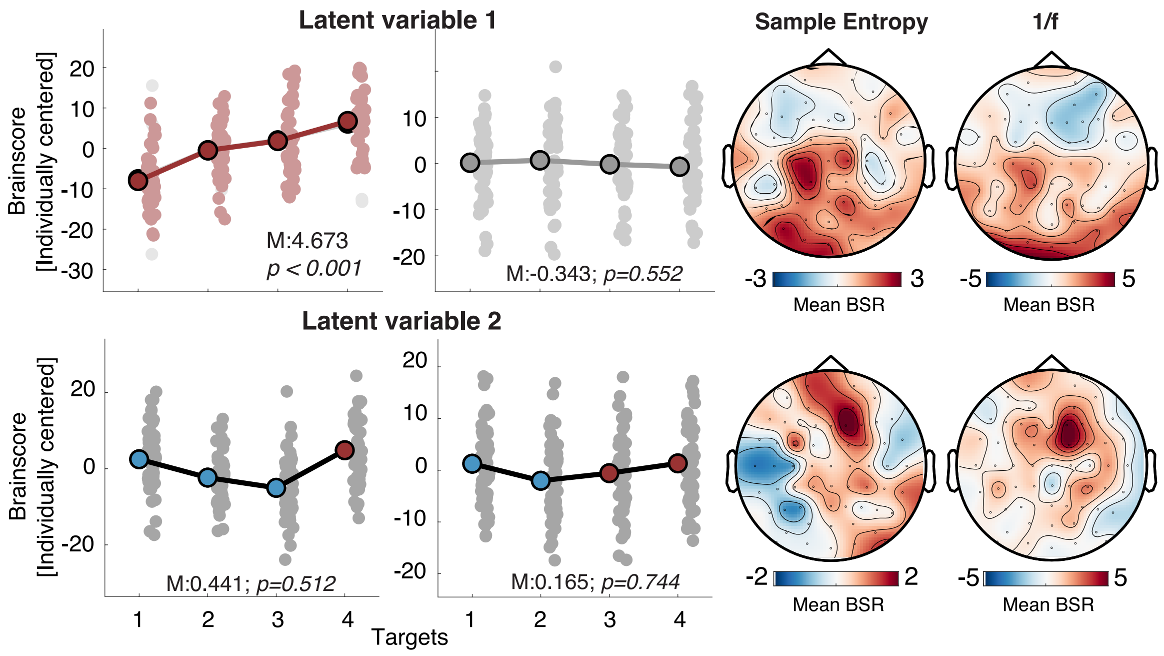

Supplementary Figure 12. Task PLS of sample entropy and aperiodic slopes across all channels. Brainscores for younger adults (N=47) are shown on the left side, with data for older adults shown to the right (N=53). Inset estimates refer to fixed linear effects models. Topographies of bootstrap ratios are unthresholded.

Supplementary Note 8

Exploratory whole-brain task PLS of aperiodic dynamics. In the main analysis, we restricted the PLS to posterior channels with the aim to predominantly characterize signals stemming from parietal and visual cortex. To explore whether this analysis missed uncertainty-related changes in aperiodic dynamics in other regions, we performed an additional task PLS analysis that included all channels. This task PLS averaged sample entropy across the final 2.5s of stimulus presentation. To normalize relative contributions of the two signatures to the PLS, we z-transformed values of each signature across target load levels prior to including them in the model. This joint PLS resulted in two significant latent variables (Supp. Fig. 12). The first latent variable (*permuted* $p = 0.001$) indicated uncertainty-related increases in sample entropy and shallowing of aperiodic slopes in younger, but not older adults. Regional contributions were predominantly observed in posterior sensors. This latent variable thus captures the observations in the main analysis. The second latent variable (*permuted* $p = 0.021$) was instead marked by quadratic changes (younger adults: $p = 1.5e\text{-}08$; older adults: $p = 0.03$; linear mixed effects model with fixed and random quadratic effects) as a function of target load. Estimates initially decreased, followed by an increase with load towards higher target load, predominantly at mediofrontal channels.

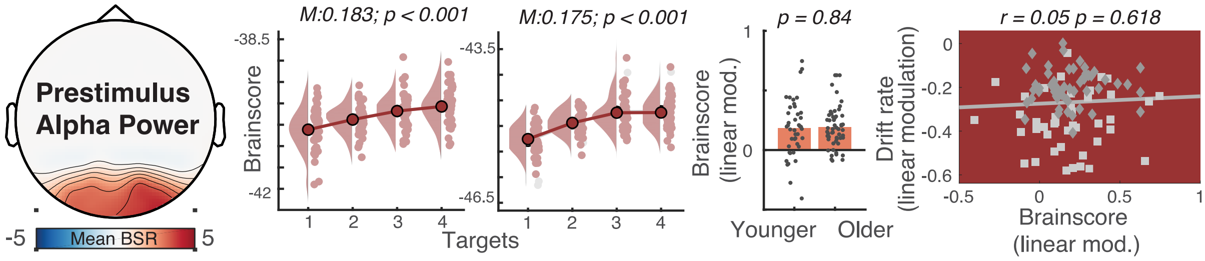

Supplementary Figure 13. Pre-stimulus alpha power. Uncertainty similarly increases pre-stimulus alpha power in younger (N=47) and older adults (N=53) but does not relate to individual drift rate adjustments. Light grey squares indicate younger adults, dark grey diamonds correspond to older adults.

Supplementary Note 9

Pre-stimulus alpha power. Evidence on age-related changes in pre-stimulus alpha power are mixed. Early studies suggest that pre-stimulus alpha synchronization (or lateralization) in the context of attentional cueing is observed exclusively for younger, but not older adults [12,13]. In contrast, [14] indicated similar pre-stimulus lateralization between age groups, whereas they noted age differences in alpha modulation during working memory retention. While our task design does not allow us to assess the lateralization of alpha power, our results indicate that pre-stimulus alpha power increases similarly alongside uncertainty in both age groups, but with no apparent relation to subsequent (delayed) task performance (Supp. Fig. 13).

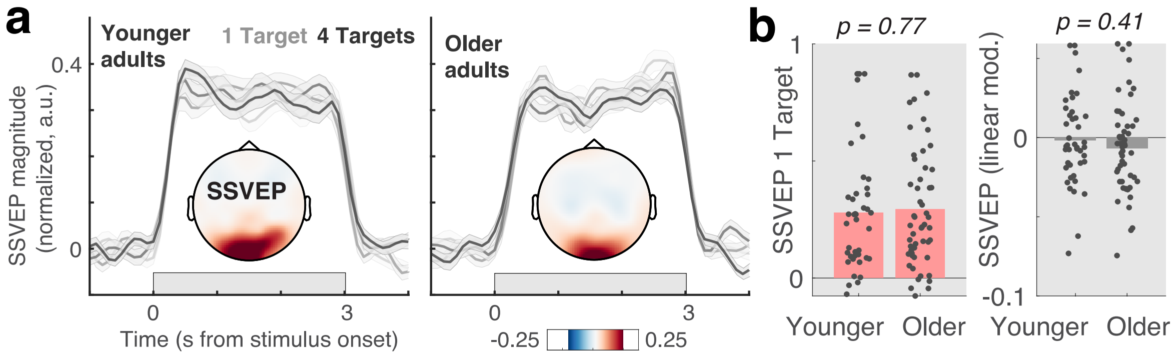

Supplementary Figure 14. Steady-state visual evoked potential (SSVEP). (a) Both age groups ($N_{younger}$ = 47, N_{older} = 53) exhibited robust SSVEPs. Time-resolved, spectrally normalized, SSVEP power, averaged across occipital channels (O1, Oz, O2), indicates clear SSVEP increases specifically during stimulus presentation. Data are presented as mean values +/- within-subject SEM. Topography insets show stimulus-evoked SSVEP contrast minus baseline. (b) However, estimates from occipital EEG channels (O1, Oz, O2) did not indicate age differences in single-target SSVEP magnitude, a main effect of load in either group, or differences in the strength of linear modulation (~ age*load interaction).

Supplementary Note 10

SSVEP magnitude. SSVEP magnitude has been suggested as a signature of encoded sensory information that is enhanced by attention [15-18] and indicates fluctuations in early visual cortex excitability [19]. However, despite a clear SSVEP signature of comparable magnitude in both younger and older adults, we did not observe significant effects of target uncertainty on SSVEP magnitude in either age group (Supp. Fig. 14). Given that the SSVEP frequency was shared across different features, we could not investigate feature selection via SSVEPs as is commonly the case in attention studies. Studies with feature-specific SSVEPs, suggest that younger adults' SSVEP magnitude differentiates between attended and unattended features, whereas no robust differentiation is observed in older adults, pointing to deficits in attentional filtering [17,18].

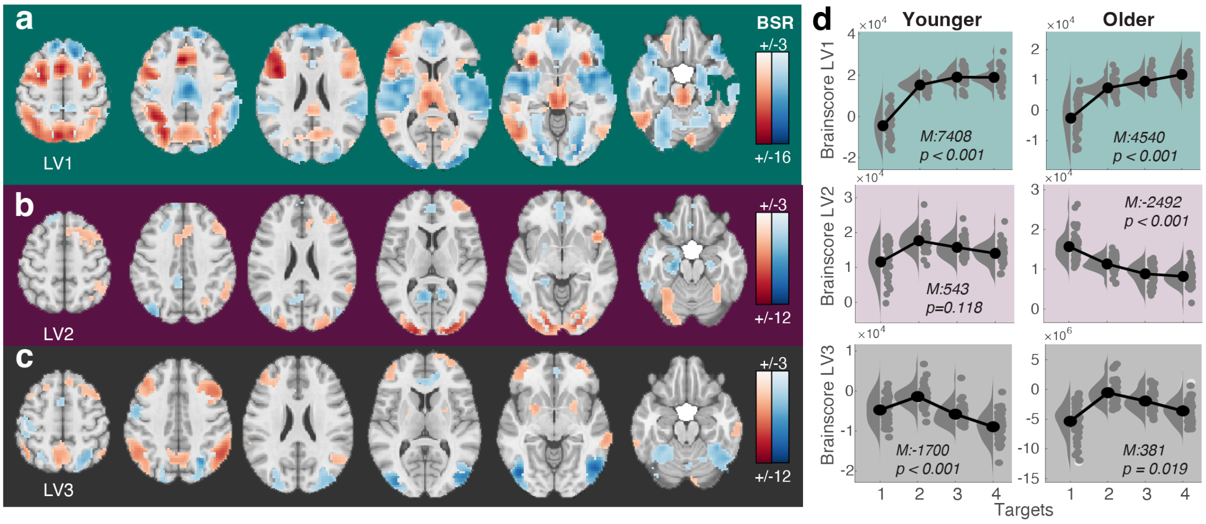

Supplementary Figure 15. Main effects of target load on BOLD magnitude. A task partial least squares (PLS) analysis indicated three significant latent variables (loadings shown in panels a-c) that were sensitive to changes in target number. (d) Statistics refer to paired t-tests of linear slopes against zero ($N_{younger}$ = 42, N_{older} = 53).

Supplementary Note 11

Main effects of target load on BOLD magnitude across the adult lifespan. We performed a whole-brain task PLS to assess potential main effects of target load on BOLD magnitude. In brief, we observed a similar first latent variable (*permuted* $p < 0.001$) to that reported in younger adults [4], highlighting uncertainty-related increases dominantly in cortical areas encompassing the frontoparietal and the midcingulo-insular network, as well as in the thalamus (see detailed results of this analysis in Supp. Fig. 15 and Tables S2-4). The task PLS indicated two further robust LVs. LV2 (*permuted* $p < 0.001$) captured a non-linear pattern in younger adults and linear changes under uncertainty in older adults. Regional contributors partly overlapped with the initial LV (Table S3). Finally, LV3 (*permuted* $p < 0.001$) captured nonlinear changes (initial increases in engagement followed by disengagement) in both age groups in a set of regions encompassing positive loadings in frontoparietal components of the executive control network, and negative loadings in temporal-occipital cortex (Table S4).

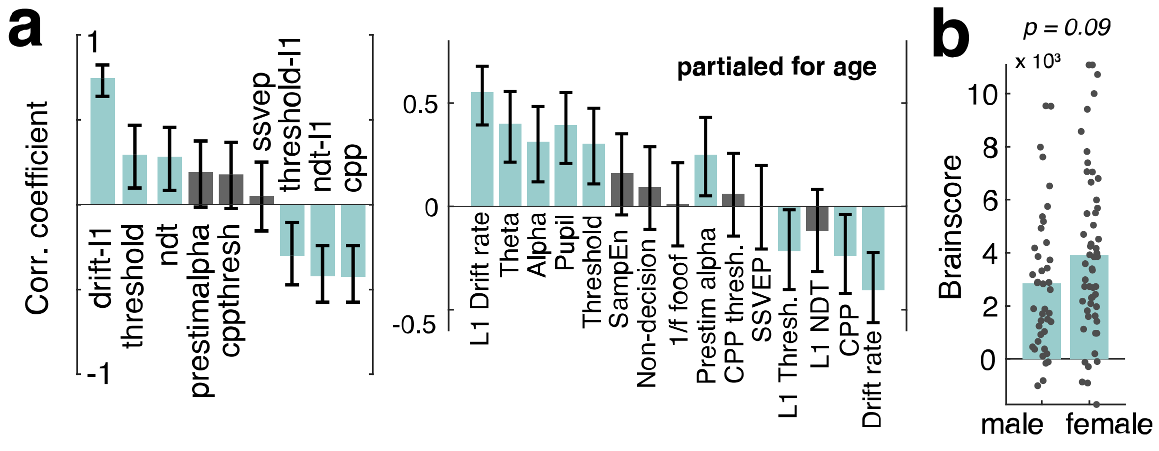

Supplementary Figure 16. Post-hoc Brainscore relations. (a) Relations before (left) and after controlling for categorical age (right). Beyond the *a priori* signatures included in the behavioral PLS model, post-hoc exploration indicated that individuals with more pronounced BOLD uncertainty modulation also had larger single-target drift rates, and lower single-target boundary separation ("boundary thresholds"), as well as larger increases in the latter as a function of uncertainty, also after controlling for categorical age (see right). In addition to the magnitude of uncertainty-related drift rate modulation, CPP slope modulation was similarly related to Brainscores. Plots indicate Pearson correlation coefficients +- 95%CI after accounting for age covariation. (b) Brainscores from behavioral PLS as a function of gender (N_{male}= 41, N_{female} = 54).

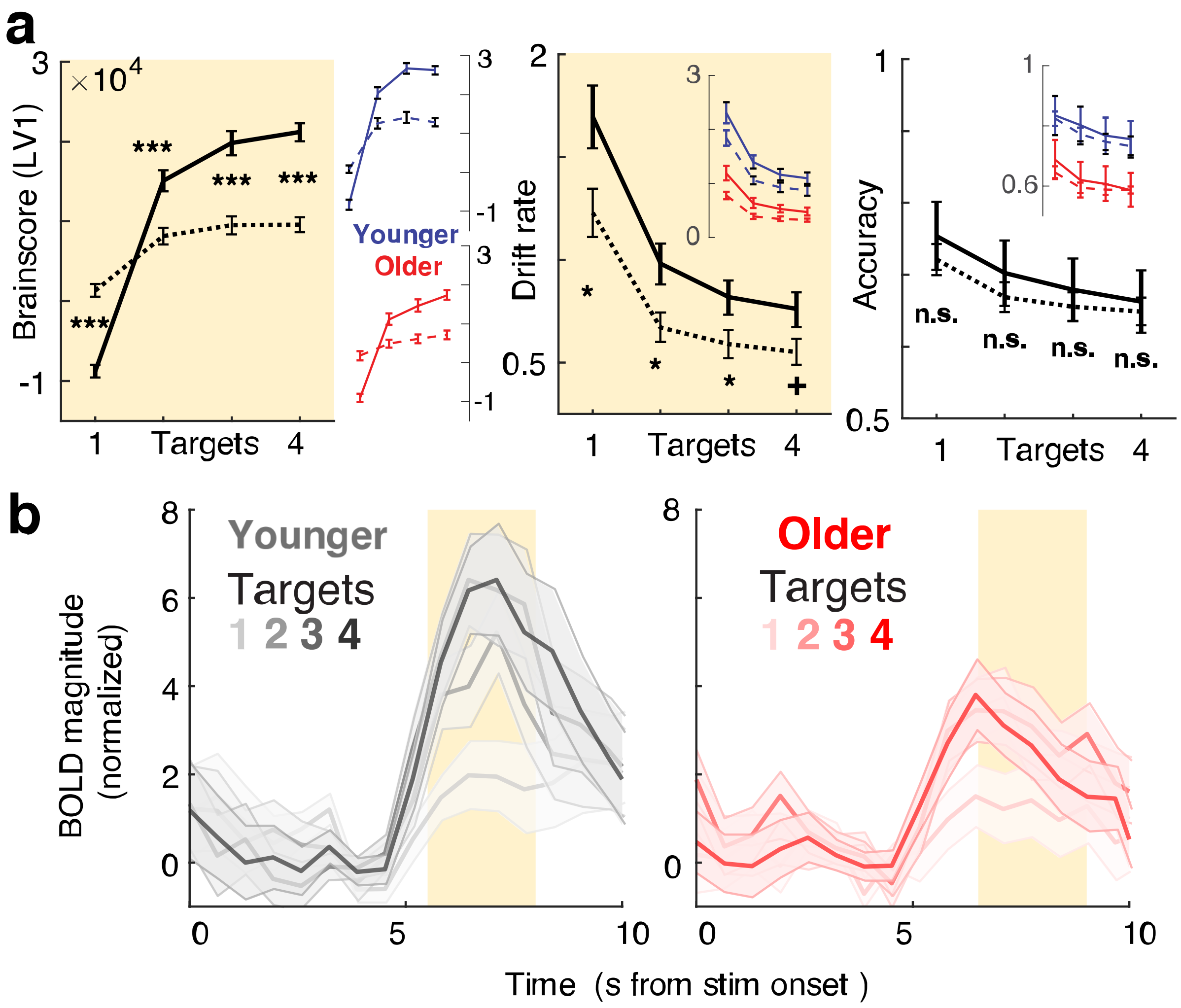

Supplementary Figure 17. BOLD modulation effects are robust to accuracy differences within (a) and between age groups (b). (a) Younger and older individuals with larger BOLD uncertainty modulation (see LV1 in Supp. Fig. 15) achieve higher drift rates across uncertainty levels at comparable accuracy levels. Data show upper (full lines) and lower (broken lines) groups of a trichotomized split of accuracy and drift rate data based on the magnitude of uncertainty change (234 vs. 1) in the 1st LV of the task PLS (closely mirroring LV1 of the behavioral PLS; N_{high}=31, N_{low}=33). Insets illustrate comparable within-group splits (split performed within-group; $N_{younger\text{-}high}$=14, $N_{younger\text{-}low}$=14, $N_{older\text{-}high}$=17, $N_{older\text{-}low}$=19). Data are means +- SEs. (b) Age x uncertainty interaction in mediodorsal thalamus for accuracy-matched features. This analysis excluded trials in which the best (YA) or worst (OA) features were probed to compare data with group-matched single-target accuracy (see **Supplementary Note 2**). A linear mixed effects model indicated a retained group x target load interaction for data averaged in the time window of interest (yellow shading; $t(364) = -2.051$, $p = 0.041$, $\beta = -0.76$, $SE = 0.37$, 95%CI = [-1.48, -0.03]). Data are means +- SEs.

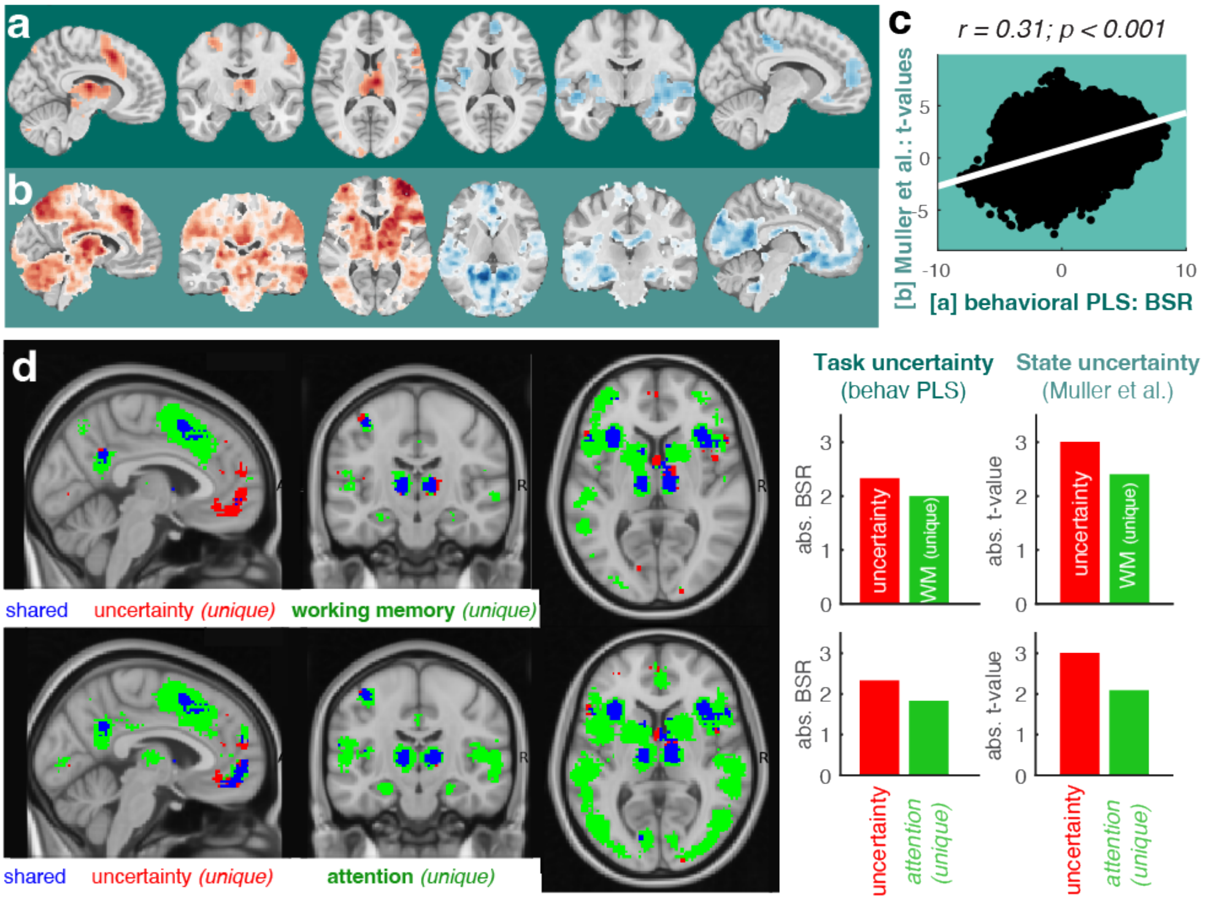

Supplementary Figure 18. Reverse inference of uncertainty, working memory, and attention. (a-c) Overlap between neuroimaging of task and state uncertainty. (a) Spatial loadings of behavioral PLS, reproduced from Fig. 6a. (b) Neuroimaging results of a state entropy manipulation with low working memory demands [reanalyzed from Fig. 1-3a in [20]; https://neurovault.org/collections/4872/]. Data range is [0 to -/+ 6]. (c) Person correlation between spatial t-values and spatial loadings indicate convergent spatial activation patterns between uncertainty manipulations (N=7299 voxels). (d) Overlap and uniqueness of uncertainty and working memory activation. Neurosynth.org activations (uniformity tests: the degree to which each voxel is consistently activated in studies that use a given term; threshold: FDR = 0.01; binarization threshold: 1) for "working memory" and "uncertainty", decomposed into shared and unique patterns. Both the current task uncertainty modulation and state uncertainty more closely resemble meta-analytic loadings of uncertainty than working memory or attention patterns.

Supplementary Note 12

Relation of fMRI modulation to uncertainty, working memory, and attention. Our uncertainty manipulation demands multifaceted adjustments, including to attentional selection, extra-dimensional set switching and working memory maintenance. Can our results be directly linked to a single cognitive concept? We performed reverse inference analyses to clarify this. In line with our results capturing variations in induced uncertainty, neuroimaging results converged with a prior assessment of model-based uncertainty (about the reward probability of eight options in a Bayesian ideal observer model [20]; Supp. Fig. 18a-c). In this prior operationalization, uncertainty varies according to varying probabilistic beliefs across features but does not directly scale with working memory load. Our results converge with raw loadings from this prior assessment (incl. on thalamic loadings not originally highlighted). When directly contrasting task uncertainty activation to meta-analytic activations to attention or working memory studies (Supp. Fig. 18d), neuroimaging results were more consistent with meta-analytic maps of uncertainty. This was also true when considering % overlap between binary empirical activation (+/-1.96 threshold) and neurosynth masks: task uncertainty: uncertainty: 53% | wm: 47% | attention: 41%; state uncertainty: uncertainty: 78% | wm: 64%| attention: 53%. Taken together, these results indicate that our results converge with prior work on uncertainty, while also indicating overlap with multifaceted executive adjustment in response to variable uncertainty.

Supplementary Note 13

Extended discussion: uncertainty and working memory capacity. Can decreasing working memory (WM) capacity with older age [21] account for dampened uncertainty adjustment? Our task consists of three stages, each of which requires WM representations. First, from cue to probe presentation, participants need to maintain 1 to 4 targets in the active task set. We aimed to reduce WM load during this phase by maintaining an identical cue set within eight-trial blocks, and re-cueing this set at the onset of each trial. Cue-related EEG responses indicate similar alpha power modulation for younger and older adults (Supp. Fig. 13), suggesting comparable processing of the cued feature set and alertness. Second, during stimulus presentation, the MAAT requires participants to concurrently sample up to four visual features and retain WM traces of feature-specific information for the upcoming probe-dependent decision phase. Third, once a probe retro-cues the decision-relevant feature, feature-specific evidence must be selected amongst competing WM representations [22]. Probe processing (and behaviour) can be affected by how many features could be encoded given individual WM capacity across latter task stages. However, behavioural (and neural) uncertainty effects (and age differences therein) were prominent also at low target loads, arguing against a major role of WM capacity constraints. Rather, the pattern of effects supports age differences in executive control over WM contents, as also observed in N-back tasks [23]. A capacity-based explanation is also less intuitive for uncertainty-induced changes at the onset of stimulus presentation (e.g., increased pupil diameter, EEG-based power). We argue that WM *fidelity* is more important in this context than WM *capacity* per se [24]. In line with this notion, our decoding analysis indicates an increase in information about multiple features at the expense of single-target fidelity. Operationalizations of task uncertainty via cue conflict (as operationalized here; see also [25,26]) are also used to probe WM fidelity under varying target predictability. Studies of the latter indicate that uncertainty reduces the precision of (probabilistic) WM representations [27,28], here theoretically for each feature (the feature-specific states shown in Fig. 7). Neuroimaging further supports a close overlap between uncertainty and WM load manipulations (see also Supp. Fig. 18): sensitivity to either relies on a "multiple demand network" [29]. Notably, mediodorsal thalamic engagement has been theorized to be maximal when multiple demands (incl. executive control operations such as WM updating, inhibition, and shifting) converge [30]. Our results therefore tacitly support the view that older adults are particularly challenged in dynamic WM management [31], and related parallel resource allocation [5], whereas WM storage capacity shows only modest age-related declines for review see [21].

Table S1: Statistics for within age-group effects. Effects were assessed via two-sided paired t-tests against zero.

Dependent variable	Fig.	df	t-value	p-value	Cohen's d
Drift rate (single-target) – younger	1b	41	26.41	2.3e-27	4.07
Drift rate (single-target) – older	1b	52	22.06	4.6e-28	3.03
CPP (single-target) – younger	1b	41	8.62	9.5e-11	1.33
CPP (single-target) – older	1b	52	7.92	1.7e-10	1.09
Drift rate (linear mod.) – younger	1b	41	-17.07	3.1e-20	-2.63
Drift rate (linear mod.) – older	1b	52	-17.45	2.2e-23	-2.4
CPP (linear mod.) – younger	1b	41	-7.37	4.9e-09	-1.14
CPP (linear mod.) – older	1b	52	-5.04	5.9e-06	-0.69
Stroop interference – younger	3b	47	10.01	3.1e-13	1.44
Stroop interference – older	3b	52	16.02	9.3e-22	2.2
Theta power (linear mod.) – younger	4a	41	6.85	2.6e-08	1.06
Theta power (linear mod.) – older	4a	52	2.3	2.6e-02	0.32
Pupil diameter (linear mod.) – younger	4b	41	7.34	5.6e-09	1.13
Pupil diameter (linear mod.) – older	4b	52	7.25	1.9e-09	1
Alpha power (linear mod.) – younger	5a	41	6.05	3.7e-07	0.93
Alpha power (linear mod.) – older	5a	52	-0.75	0.46	-0.1
Sample Entropy (linear mod.) – younger	5b	41	2.21	0.033	0.34
Sample Entropy (linear mod.) – older	5b	52	0.23	0.82	0.03
1/f slope (linear mod.) – younger	5c	41	4.67	3.3e-05	0.72
1/f slope (linear mod.) – older	5c	52	0.14	0.89	0.02
fMRI Brainscore – younger	6b	41	11.49	2.1e-14	1.77
fMRI Brainscore – older	6b	52	7.47	8.8e-10	1.03

Table S2: PLS model peak activations, bootstrap ratios, and cluster sizes for task PLS LV1.

		MNI Coordinates				
Region	Hem	X	Y	Z	BSR	#Voxels
IFG (p. Opercularis)	L	-45	9	27	15.11	3164
Inferior Parietal Lobule	L	-42	-48	45	14.3	3451
Insula Lobe	R	30	21	-3	11.4	170
Inferior Temporal Gyrus	L	-54	-66	-12	11.34	880
Thalamus	L	-6	-30	-3	10.76	1064
Superior Frontal Gyrus	R	27	-3	54	10.21	903
Cerebelum (Crus 1)	R	6	-81	-24	8.5	276
Cerebelum (VI)	R	30	-66	-30	7.83	129
Inferior Temporal Gyrus	R	54	-63	-12	6.19	297
Area Fo3	L	-27	39	-21	4.68	45
Calcarine Gyrus	L	-15	-78	6	4.28	29
Middle Frontal Gyrus	R	27	51	3	4.25	31
Superior Medial Gyrus	R	12	48	33	-12.32	2317
Area hOc3d [V3d]	L	-24	-99	12	-11.64	6542
MCC	R	3	-15	36	-11.23	889
Area lg1	R	30	-21	3	-11.14	4238
Postcentral Gyrus	R	21	-36	63	-5.86	121
Postcentral Gyrus	L	-39	-21	36	-5.8	43
Middle Frontal Gyrus	L	-33	24	39	-5.32	32
Angular Gyrus	L	-48	-63	27	-4.81	59
Middle Frontal Gyrus	R	45	15	48	-3.95	55

Table S3: PLS model peak activations, bootstrap ratios, and cluster sizes for task PLS LV2.

		MNI Coordinates				
Region	Hem	X	Y	Z	BSR	#Voxels
Area hOc3d [V3d]	L	-24	-99	12	9.16	1974
Insula Lobe	R	42	15	-3	6.66	136
Middle Orbital Gyrus	R	30	54	-15	5.31	143
Superior Frontal Gyrus	R	21	12	60	5.01	911
Middle Frontal Gyrus	R	33	48	12	4.91	119
Angular Gyrus	R	57	-51	27	4.76	280
Inferior Temporal Gyrus	R	51	-3	-39	4.47	50
Inferior Temporal Gyrus	R	63	-27	-30	4.4	28
Superior Occipital Gyrus	R	21	-63	42	4.05	69
Rolandic Operculum	L	-57	6	3	3.79	27
Hippocampus	L	-27	-21	-18	-7.36	316
Calcarine Gyrus	L	-12	-60	12	-6.5	244
Rectal Gyrus	L	-9	27	-15	-6.24	440
Middle Temporal Gyrus	L	-66	-57	-9	-6.12	256
Middle Occipital Gyrus	L	-42	-81	39	-6.08	190
IFG (p. Orbitalis)	L	-36	33	-18	-5.97	94
Precuneus	R	9	-54	9	-5.67	131
ParaHippocampal Gyrus	R	21	-21	-18	-4.83	39
IFG (p. Orbitalis)	R	24	27	-15	-4.67	28
Middle Temporal Gyrus	R	54	-6	-15	-4.56	51
MCC	L	-12	-42	36	-4.44	76
Middle Occipital Gyrus	R	45	-78	27	-4.34	34
Middle Frontal Gyrus	L	-27	30	42	-4.27	153
Cerebelum (Crus 2)	R	3	-87	-33	-4.14	28
Middle Temporal Gyrus	L	-51	-3	-24	-3.94	53
Superior Frontal Gyrus	L	-24	60	3	-3.78	27

Table S4: PLS model peak activations, bootstrap ratios, and cluster sizes for task PLS LV3.

		MNI Coordinates				
Region	Hem	X	Y	Z	BSR	#Voxels
Angular Gyrus	R	51	-57	36	8.05	604
Middle Frontal Gyrus	R	36	21	36	6.87	660
Inferior Parietal Lobule	L	-54	-51	42	6.27	469
Precuneus	L	-9	-66	45	6.13	474
Middle Frontal Gyrus	L	-39	24	33	6.05	726
Middle Frontal Gyrus	R	27	57	0	5.97	287
Middle Temporal Gyrus	R	60	-33	-12	5.46	184
Cerebelum (Crus 1)	R	9	-81	-27	5.29	62
Putamen	L	-27	6	-6	4.82	74
Putamen	R	24	0	6	4.38	67
Inferior Temporal Gyrus	L	-66	-42	-21	4.16	49
Cerebelum (Crus 2)	L	-15	-87	-30	3.96	32
Cerebelum (Crus 2)	R	33	-72	-45	3.8	53
Inferior Temporal Gyrus	R	48	-69	-9	-10.33	1706
Inferior Occipital Gyrus	L	-45	-75	-6	-9.9	1022
Postcentral Gyrus	L	-57	-6	39	-5.18	232
Postcentral Gyrus	L	-51	-33	57	-4.5	43
ACC	R	12	42	9	-4.41	191
Superior Parietal Lobule	L	-24	-63	48	-4.4	36
Posterior-Medial Frontal	L	-6	3	57	-4.4	65

Table S5: PLS model peak activations, bootstrap ratios, and cluster sizes for behavioral PLS LV1.

		MNI Coordinates				
Region	Hem	X	Y	Z	BSR	#Voxels
Thalamus	L	-6	-15	12	8.57	573
Posterior-Medial Frontal	L	3	12	45	8.13	555
Precentral Gyrus	L	-42	0	30	7.51	931
Superior Frontal Gyrus	R	27	-3	54	6.91	222
Inferior Occipital Gyrus	L	-42	-72	-6	6.13	208
Middle Temporal Gyrus	L	-51	-51	21	6.03	90
Putamen	R	30	18	0	5.75	94
Middle Frontal Gyrus	R	36	24	21	5.74	220
Middle Temporal Gyrus	L	-57	-33	-6	5.26	35
Inferior Parietal Lobule	R	30	-54	48	5.14	323
Inferior Parietal Lobule	L	-36	-57	45	4.75	315
Area hOc1 [V1]	R	9	-99	6	4.7	82
Inferior Temporal Gyrus	R	45	-63	-12	4.51	27
Hippocampus	L	-27	-18	-21	-8.92	873
MCC	L	-12	-36	48	-6.05	359
Putamen	R	30	-3	9	-5.79	879
Superior Frontal Gyrus	R	18	54	30	-5.73	443
Middle Frontal Gyrus	L	-21	30	54	-5.45	67
Superior Medial Gyrus	R	12	42	48	-5.28	138
IFG (p. Orbitalis)	R	36	30	-21	-5.2	55
ParaHippocampal Gyrus	R	21	-18	-18	-5.13	49
Middle Temporal Gyrus	L	-51	3	-33	-4.28	29
Inferior Frontal Gyrus	L	-33	36	-21	-4.17	26
Rectal Gyrus	L	-9	24	-12	-4.15	73
Inferior Temporal Gyrus	L	-57	-24	-27	-4.03	32

Supplementary References

1 Starns, J. J. & Ratcliff, R. Age-related differences in diffusion model boundary optimality with both trial-limited and time-limited tasks. *Psychon Bull Rev* **19**, 139-145 (2012). https://doi.org:10.3758/s13423-011-0189-3

2 Starns, J. J. & Ratcliff, R. The effects of aging on the speed-accuracy compromise: Boundary optimality in the diffusion model. *Psychology and aging* **25**, 377-390 (2010). https://doi.org:10.1037/a0018022

3 McGovern, D. P., Hayes, A., Kelly, S. P. & O'Connell, R. G. Reconciling age-related changes in behavioural and neural indices of human perceptual decision-making. *Nat Hum Behav* **2**, 955-966 (2018). https://doi.org:10.1038/s41562-018-0465-6

4 Kosciessa, J. Q., Lindenberger, U. & Garrett, D. D. Thalamocortical excitability modulation guides human perception under uncertainty. *Nat Commun* **12**, 2430 (2021). https://doi.org:10.1038/s41467-021-22511-7

5 Jaroslawska, A. J. *et al.* What affects the magnitude of age-related dual-task costs in working memory? The role of stimulus domain and access to semantic representations. *Quarterly journal of experimental psychology* **74**, 682-704 (2021). https://doi.org:10.1177/1747021820970744

6 Frömer, R., Nassar, M. R., Ehinger, B. V. & Shenhav, A. Common neural choice signals can emerge artefactually amid multiple distinct value signals. *Nature Human Behaviour* (2024). https://doi.org:10.1038/s41562-024-01971-z

7 Ehinger, B. V. & Dimigen, O. Unfold: an integrated toolbox for overlap correction, non-linear modeling, and regression-based EEG analysis. *PeerJ* **7**, e7838 (2019). https://doi.org:10.7717/peerj.7838

8 O'Connell, R. G., Dockree, P. M. & Kelly, S. P. A supramodal accumulation-to-bound signal that determines perceptual decisions in humans. *Nat Neurosci* **15**, 1729-1735 (2012). https://doi.org:10.1038/nn.3248

9 Twomey, D. M., Murphy, P. R., Kelly, S. P. & O'Connell, R. G. The classic P300 encodes a build-to-threshold decision variable. *The European journal of neuroscience* **42**, 1636-1643 (2015). https://doi.org:10.1111/ejn.12936

10 Churchland, A. K., Kiani, R. & Shadlen, M. N. Decision-making with multiple alternatives. *Nat Neurosci* **11**, 693-702 (2008). https://doi.org:10.1038/nn.2123

11 Donner, T. H., Siegel, M., Fries, P. & Engel, A. K. Buildup of choice-predictive activity in human motor cortex during perceptual decision making. *Current biology : CB* **19**, 1581-1585 (2009). https://doi.org:10.1016/j.cub.2009.07.066

12 Vaden, R. J., Hutcheson, N. L., McCollum, L. A., Kentros, J. & Visscher, K. M. Older adults, unlike younger adults, do not modulate alpha power to suppress irrelevant information. *Neuroimage* **63**, 1127-1133 (2012). https://doi.org:10.1016/j.neuroimage.2012.07.050

13 Hong, X. F., Sun, J. F., Bengson, J. J., Mangun, G. R. & Tong, S. B. Normal aging selectively diminishes alpha lateralization in visual spatial attention. *Neuroimage* **106**, 353-363 (2015). https://doi.org:10.1016/j.neuroimage.2014.11.019

14 Leenders, M. P., Lozano-Soldevilla, D., Roberts, M. J., Jensen, O. & De Weerd, P. Diminished Alpha Lateralization During Working Memory but Not During Attentional Cueing in Older Adults. *Cereb Cortex* **28**, 21-32 (2018). https://doi.org:10.1093/cercor/bhw345

15 Morgan, S. T., Hansen, J. C. & Hillyard, S. A. Selective attention to stimulus location modulates the steady-state visual evoked potential. *Proceedings of the National Academy of Sciences of the United States of America* **93**, 4770-4774 (1996). https://doi.org:10.1073/pnas.93.10.4770

16 Muller, M. M. *et al.* Feature-selective attention enhances color signals in early visual areas of the human brain. *Proceedings of the National Academy of Sciences of the United States of America* **103**, 14250-14254 (2006). https://doi.org:10.1073/pnas.0606668103

17 Quigley, C. & Muller, M. M. Feature-selective attention in healthy old age: a selective decline in selective attention? *The Journal of neuroscience : the official journal of the Society for Neuroscience* **34**, 2471-2476 (2014). https://doi.org:10.1523/JNEUROSCI.2718-13.2014

18 Quigley, C., Andersen, S. K., Schulze, L., Grunwald, M. & Muller, M. M. Feature-selective attention: evidence for a decline in old age. *Neuroscience letters* **474**, 5-8 (2010). https://doi.org:10.1016/j.neulet.2010.02.053

19 Zhigalov, A., Herring, J. D., Herpers, J., Bergmann, T. O. & Jensen, O. Probing cortical excitability using rapid frequency tagging. *Neuroimage* **195**, 59-66 (2019). https://doi.org:10.1016/j.neuroimage.2019.03.056

20 Muller, T. H., Mars, R. B., Behrens, T. E. & O'Reilly, J. X. Control of entropy in neural models of environmental state. *Elife* **8** (2019). https://doi.org:10.7554/eLife.39404

21 Naveh-Benjamin, M. & Cowan, N. The roles of attention, executive function and knowledge in cognitive ageing of working memory. *Nat Rev Psychol* **2**, 151-165 (2023). https://doi.org:10.1038/s44159-023-00149-0

22 Mok, R. M., Myers, N. E., Wallis, G. & Nobre, A. C. Behavioral and Neural Markers of Flexible Attention over Working Memory in Aging. *Cereb Cortex* **26**, 1831-1842 (2016). https://doi.org:10.1093/cercor/bhw011

23 Bopp, K. L. & Verhaeghen, P. Aging and n-Back Performance: A Meta-Analysis. *The journals of gerontology. Series B, Psychological sciences and social sciences* **75**, 229-240 (2020). https://doi.org:10.1093/geronb/gby024

24 Hill, P. F., King, D. R. & Rugg, M. D. Age Differences In Retrieval-Related Reinstatement Reflect Age-Related Dedifferentiation At Encoding. *Cereb Cortex* **31**, 106-122 (2021). https://doi.org:10.1093/cercor/bhaa210

25 Mukherjee, A., Lam, N. H., Wimmer, R. D. & Halassa, M. M. Thalamic circuits for independent control of prefrontal signal and noise. *Nature* (2021). https://doi.org:10.1038/s41586-021-04056-3
26 Tsumura, K. *et al.* Reversible Fronto-occipitotemporal Signaling Complements Task Encoding and Switching under Ambiguous Cues. *Cereb Cortex* **32**, 1911-1931 (2022). https://doi.org:10.1093/cercor/bhab324
27 Li, H. H., Sprague, T. C., Yoo, A. H., Ma, W. J. & Curtis, C. E. Joint representation of working memory and uncertainty in human cortex. *Neuron* **109**, 3699-3712 e3696 (2021). https://doi.org:10.1016/j.neuron.2021.08.022
28 Ma, W. J., Husain, M. & Bays, P. M. Changing concepts of working memory. *Nat Neurosci* **17**, 347-356 (2014). https://doi.org:10.1038/nn.3655
29 Duncan, J. The multiple-demand (MD) system of the primate brain: mental programs for intelligent behaviour. *Trends in cognitive sciences* **14**, 172-179 (2010). https://doi.org:10.1016/j.tics.2010.01.004
30 Pergola, G. *et al.* The Regulatory Role of the Human Mediodorsal Thalamus. *Trends in cognitive sciences* **22**, 1011-1025 (2018). https://doi.org:10.1016/j.tics.2018.08.006
31 Zuber, S. *et al.* Explaining age differences in working memory: The role of updating, inhibition, and shifting. *Psychology & Neuroscience* **12**, 191-208 (2019). https://doi.org:10.1037/pne0000151
