# Supplementary material for: Broadscale dampening of uncertainty adjustment in the aging brain
Source: Nat Commun. 2024 Dec 23;15:10717. doi: 10.1038/s41467-024-55416-2 (PMC11666723; doi:10.1038/s41467-024-55416-2)
Supplement: Supplementary file 3 — Reporting Summary [file 41467_2024_55416_MOESM3_ESM.pdf]

nature portfolio

Corresponding author(s): Julian Kosciessa, Douglas Garrett

Last updated by author(s): Dec 4, 2024

Reporting Summary

Nature Portfolio wishes to improve the reproducibility of the work that we publish. This form provides structure for consistency and transparency in reporting. For further information on Nature Portfolio policies, see our Editorial Policies and the Editorial Policy Checklist.

Statistics

For all statistical analyses, confirm that the following items are present in the figure legend, table legend, main text, or Methods section.

n/a	Confirmed	
☐	☒	The exact sample size (*n*) for each experimental group/condition, given as a discrete number and unit of measurement
☐	☒	A statement on whether measurements were taken from distinct samples or whether the same sample was measured repeatedly
☐	☒	The statistical test(s) used AND whether they are one- or two-sided<br>*Only common tests should be described solely by name; describe more complex techniques in the Methods section.*
☐	☒	A description of all covariates tested
☐	☒	A description of any assumptions or corrections, such as tests of normality and adjustment for multiple comparisons
☐	☒	A full description of the statistical parameters including central tendency (e.g. means) or other basic estimates (e.g. regression coefficient) AND variation (e.g. standard deviation) or associated estimates of uncertainty (e.g. confidence intervals)
☐	☒	For null hypothesis testing, the test statistic (e.g. *F*, *t*, *r*) with confidence intervals, effect sizes, degrees of freedom and *P* value noted<br>*Give P values as exact values whenever suitable.*
☐	☒	For Bayesian analysis, information on the choice of priors and Markov chain Monte Carlo settings
☐	☒	For hierarchical and complex designs, identification of the appropriate level for tests and full reporting of outcomes
☐	☒	Estimates of effect sizes (e.g. Cohen's *d*, Pearson's *r*), indicating how they were calculated

Our web collection on statistics for biologists contains articles on many of the points above.

Software and code

Policy information about availability of computer code

Data collection	BrainVisionRecorder<br>PsychToolbox 3.0.11<br>Custom experiment code is available from https://doi.org/10.5281/zenodo.14216065.
Data analysis	MATLAB R2016B, R2017B<br>FieldTrip 20170904<br>SPM 12<br>PLS toolbox v.6.15 (incl. custom adaptations for recent MATLAB versions available upon request)<br>MEG-PLS toolbox [version 2.02b]<br>mMSE (available from https://github.com/LNDG/mMSE); SPM Anatomy Toolbox (Version 2.2c)<br>FSL 5 (RRID:SCR_002823)<br>ANTs 2.1.0<br>EyeLink v4.40<br>MEG-PLS v2.02b<br>R 4.0.3<br>HDDM v.0.6.0<br><br>All analysis code is available within nested DataLad datasets from https://doi.org/10.5281/zenodo.14221999. Where possible, version controlled software packages have been linked as submodules to the relevant analyses.

For manuscripts utilizing custom algorithms or software that are central to the research but not yet described in published literature, software must be made available to editors and reviewers. We strongly encourage code deposition in a community repository (e.g. GitHub). See the Nature Portfolio guidelines for submitting code & software for further information.

Data

Policy information about availability of data

All manuscripts must include a data availability statement. This statement should provide the following information, where applicable:

- Accession codes, unique identifiers, or web links for publicly available datasets
- A description of any restrictions on data availability
- For clinical datasets or third party data, please ensure that the statement adheres to our policy

The raw EEG, fMRI, and behavioral data generated in this study have been deposited as DataLad datasets under https://doi.org/10.5281/zenodo.14264868.

Structural MRI data are available under restricted access for data privacy reasons as per obtained informed consent. Defaced structural MRI data can be obtained after signing an access agreement with Research Data Management (rdm@mpib-berlin.mpg.de). In the access request, please specify your research aims and submit a signed data protection statement (that can be found in the BIDS directory). We aim to process requests within a month.

Research involving human participants, their data, or biological material

Policy information about studies with human participants or human data. See also policy information about sex, gender (identity/presentation), and sexual orientation and race, ethnicity and racism.

Reporting on sex and gender	Gender of participants was determined based on self-report. The study design aimed at equal sample sizes of male and female participants in either age group. A control analysis of gender on the main outcome was performed and reported.
Reporting on race, ethnicity, or other socially relevant groupings	Data on race, ethnicity, or other socially relevant groupings was not collected.
Population characteristics	47 healthy young adults (mean age = 25.8 years, SD = 4.6, range 18 to 35 years; 25 women) and 53 healthy older adults (mean age = 68.7 years, SD = 4.2, range 59 to 78 years; 28 women) participated in the EEG session. 42 younger adults and all older adults returned for a subsequent 3T fMRI session. Gender of participants was determined based on self-report. Participants were recruited from the participant database of the Max Planck Institute for Human Development, Berlin, Germany (MPIB). Participants were right-handed, as assessed with a modified version of the Edinburgh Handedness Inventory 160, and had normal or corrected-to-normal vision. Participants reported to be in good health with no known history of neurological or psychiatric incidences All older adults had Mini Mental State Examination (MMSE) scores above 25.
Recruitment	Participants were randomly recruited via telephone calls using the participant database at the Max Planck Institute for Human Development. Participants needed to be in good health to fulfill the internal criteria for EEG and fMRI studies. Participant recruitment was performed by a team that did not include the principal researchers, and participants were blind to the specific study hypotheses. While we do not perceive a major self-selection bias to participate in the study beyond a general interest in cognitive psychology/neuroscience studies, the sample of older adults may on average have a higher education level and health status (due to the need to pass the inclusion requirements for EEG and fMRI) compared to the general population.
Ethics oversight	The ethics board of the Deutsche Gesellschaft für Psychologie (DGPs) approved the study protocol (“Flexibles Denken - State Switch”). All participants gave their written informed consent prior to participating in the study.

Note that full information on the approval of the study protocol must also be provided in the manuscript.

Field-specific reporting

Please select the one below that is the best fit for your research. If you are not sure, read the appropriate sections before making your selection.

☒ Life sciences ☐ Behavioural & social sciences ☐ Ecological, evolutionary & environmental sciences

For a reference copy of the document with all sections, see nature.com/documents/nr-reporting-summary-flat.pdf

Life sciences study design

All studies must disclose on these points even when the disclosure is negative.

Sample size	We recruited a total of N = 100 participants, with approximately age-matched sample sizes. A group size of ca. N=50 per age group was based on sufficient statistical power in our prior work in younger adults (Kosciessa et al., 2021, Nat. Comms.; target N = 50 prior to dropouts).
Data exclusions	No data were excluded from the analyses. N = 5 younger adults dropped out following the initial EEG session with no reasons given. Analyses are based on the full available sample for each modality.
Replication	No independent replication was attempted in the present manuscript. Reproducibility of results from final analyses were successfully verified by the first author. Where possible, specific seeds have been used to increase the reproducibility of stochastic algorithms, and results from stochastic algorithms have been stored. To facilitate independent reproducibility attempts, DataLad datasets containing comprehensive code,

	data and tools used for all reported analyses are provided under https://gin.g-node.org/StateSwitch/stsw (see also https://doi.org/10.5281/zenodo.14221999). Please contact the corresponding authors for access to processed data from specific analyses.
Randomization	No allocation to experimental groups was undertaken at the level of subjects. Across experimental conditions, presentation was pseudo-randomized such that every size and constellation of the cue set was presented across blocks. Within each run of four blocks, every set size was presented once, but never directly following a block of the same set size. In every block, each feature in the active set acted as a probe in at least one trial. Moreover, any attribute equally often served as a probe across all blocks. Winning options for each feature were balanced across trials, such that (correct) button responses were equally distributed across the experiment.
Blinding	No group allocation was performed in this study, thus no blinding was performed

Reporting for specific materials, systems and methods

We require information from authors about some types of materials, experimental systems and methods used in many studies. Here, indicate whether each material, system or method listed is relevant to your study. If you are not sure if a list item applies to your research, read the appropriate section before selecting a response.

Materials & experimental systems

n/a	Involved in the study
☒	☐ Antibodies
☒	☐ Eukaryotic cell lines
☒	☐ Palaeontology and archaeology
☒	☐ Animals and other organisms
☒	☐ Clinical data
☒	☐ Dual use research of concern
☒	☐ Plants

Methods

n/a	Involved in the study
☒	☐ ChIP-seq
☒	☐ Flow cytometry
☐	☒ MRI-based neuroimaging

Plants

Seed stocks	NA
Novel plant genotypes	NA
Authentication	NA

Magnetic resonance imaging

Experimental design

Design type	task; event-related
Design specifications	In one MRI session (and an additional EEG session, participants performed 4 runs a 32 blocks containing 8 trials, i.e., a total of 256 trials. Each trial was structured as follows: cue onset during which the relevant targets were centrally presented (1 s), fixation phase (2 s), dynamic stimulus phase (3 s), probe phase (incl. response; 2 s); ITI (un-jittered; 1.5 s). The current analyses concern the dynamic stimulus phase.
Behavioral performance measures	Variables: button press (left, right; recoded as correct/incorrect), reaction time. Mean accuracy was analyzed to establish that subjects performed the task as expected.

Acquisition

Imaging type(s)	structural, functional
Field strength	3 Tesla
Sequence & imaging parameters	Whole-brain task fMRI data (4 runs á ~11,5 mins, 1066 volumes per run) were collected via a 3T Siemens TrioTim MRI system (Erlangen, Germany) using a multi-band EPI sequence (factor 4; TR = 645 ms; TE = 30 ms; flip angle 60°; FoV = 222 mm; voxel size 3x3x3 mm; 40 transverse slices. The first 12 volumes (12 × 645 ms = 7.7 sec) were removed to ensure a steady state of tissue magnetization (total remaining volumes = 1054 per run). A T1-weighted structural scan was also acquired (MPRAGE: TR = 2500 ms; TE = 4.77 ms; flip angle 7°; FoV = 256 mm; voxel size 1x1x1 mm; 192 sagittal

slices). A T2-weighted structural scan was also acquired (GRAPPA: TR = 3200 ms; TE = 347 ms; FoV = 256 mm; voxel size 1x1x1 mm; 176 sagittal slices).

Area of acquisition: whole-brain

Diffusion MRI: ☐ Used ☒ Not used

Preprocessing

Preprocessing software: FSL 5 (RRID:SCR_002823): McFLIRT, 7 mm smoothing, .01 Hz high-pass filtering (8th order zero-phase Butterworth filter)

Normalization: We registered individual functional runs to the individual, ANTs brain-extracted T2w images (6 DOF), to T1w images (6 DOF). Finally, those images were normalized to 3mm standard space (ICBM 2009c MNI152 nonlinear symmetric) using nonlinear transformations in ANTs.

Normalization template: ICBM 2009c MNI152 nonlinear symmetric

Noise and artifact removal: Noise and artifacts from non-neural sources were identified based on visual inspection of independent components. Independent Component Analysis (ICA) was performed using FSL-MELODIC and noise components were manually identified via visual inspection. Labeling criteria are described in the manuscript. 6 DOF motion parameters, avg. white matter and CSF signal were regressed from the data. Nuisance regressors were included in the 1st level analysis (24 motion parameters, DVARS estimates).

Volume censoring: DVARS outliers (Afyouni & Nichols, 2018) were censored and interpolated as described in Power et al. (2014) and Parkes et al. (2018)

Statistical modeling & inference

Model type and settings: 1st level mass univariate (SPM) model: beta weights of stimulus onset BOLD response for each cue set size (autoregressive modelling: FAST; canonical HRF; 24 motion + DVARS + HRF derivative as nuisance regressors); 2nd level: multivariate relation between 1st level beta weights (see below)

Effect(s) tested: (1) Task PLS: main effect of cue set size; (2) Relation of linear set-size-related changes in 1st level betas to independent variables of interest (behavior, EEG, pupil, see below)

Specify type of analysis: ☐ Whole brain ☐ ROI-based ☒ Both

Anatomical location(s): Thalamic parcels were selected based on existing parcellation schemes

Statistic type for inference (See Eklund et al. 2016): Cluster-wise inference at 2nd level; cluster definition: minimum distance of 10 mm; size threshold: 25 voxels; statistical threshold: PLS bootstrap ratio > 3 (=>99.5% threshold)

Correction: permutation correction within PLS toolbox (see McIntosh & Lobaugh, 2004)

Models & analysis

n/a	Involved in the study
☒	☐ Functional and/or effective connectivity
☒	☐ Graph analysis
☐	☒ Multivariate modeling or predictive analysis

Multivariate modeling and predictive analysis: We performed a multivariate partial least squares (PLS) analysis across subjects with the following independent variables: drift-diffusion estimates (drift, threshold, non-decision time; baseline and modulation), spectral power modulation factor (EEG), spectral slope modulation (EEG), sample entropy modulation (EEG), pupil modulation.

We also performed a support vector-based analysis to decode prevalent feature options that were shown to participants (see methods).
